# Hysterosalpingography Finding in Intra Uterine Adhesion (Asherman’ s Syndrome): A Pictorial Essay

**Published:** 2013-09-18

**Authors:** Firoozeh Ahmadi, Shiva Siahbazi, Farnaz Akhbari, Bita Eslami, Ahmad Vosough

**Affiliations:** Department of Reproductive Imaging at Reproductive Biomedicine Research Center, Royan Institute for Reproductive Biomedicine, ACECR, Tehran, Iran

**Keywords:** Hysterosalpingography, Adhesion, Asherman’s Syndrome, Imaging Technique

## Abstract

Destruction of the endometrium due to trauma to the basal layer of endometrium may cause intra uterine adhesions, known as Asherman’s syndrome (AS). There are various types of imaging method for diagnosis of the intra uterine adhesion such as hysterosalpingography, sonohysterography, ultrasonography, and hysteroscopy which is considered as the gold standard approach. Hysterosalpingogram may suggest the presence of intrauterine adhesions, and may reveal the extent of the scar formation. Knowing different images in each technique is helpful in detection of intra uterine adhesion.

## Introduction

Asherman’s syndrome (AS) was first described by Heinrich Fritsch in 1894 (1), then it was further characterized by a gynecologist, Joseph Asherman, in 1950 (2). This acquired uterine condition is characterized with a wide range of partial adhesions (occurring in some part of the uterus) to complete adhesion (front and back walls of the uterus stick to one another) within the uterine cavity due to scars. It is also termed as follows: uterine synechiae, intrauterine adhesions (IUA), uterine/cervical atresia, traumatic uterine atrophy and sclerotic endometrioma (3).

Trauma to the basal layer due to dilation and curettage (D&C), after a miscarriage, delivery and medical abortion are the most common predisposing factors for AS (4). Other factors causing this condition are as follows: pelvic surgery such as cesarean section and myomectomy, intra uterine devices (IUDs), pelvic infection such as schistosomiasis and genital tuberculosis, as well as after mullerian anomalies surgery (5). Infection even in low-grade or subclinical case is always associated with scarring (6). About 40% of patients undergoing repeated D&C for retaining products of conception after missed abortion or retaining placenta (7, 8), and 25% of D&Cs which is performed within 1-4 weeks post-partum (9, 10) develop the risk of AS. Some studies have reported that the risk of AS is 16% after one D&C, while it is 32% after three or more D&Cs (11).

There are various classification systems to describe AS. Classification systems have been developed to describe the location and severity of adhesions inside the uterus. For instance, mild cases with adhesions restricted to the cervix may present with amenorrhea and infertility, it shows that symptoms alone do not indicate the severity of condition.

Early diagnosis and appropriate treatment by the removal of adhesion improve reproductive outcome of infertile women and resolve abnormal uterine bleeding (AUB) complications. According to the American Society of Reproductive Medicine (ASRM), the type and severity of the adhesions correlates with the two following reproductive outcomes: i. After removing mild to moderate uterine adhesions, patient has 70 to 80% full-term pregnancy success rates, while normal menstruation is restored in over 90% of patients (12, 13), ii. If the intrauterine adhesions are severe or cause extensive damage to the endometrial lining, fullterm pregnancy success rates are only 20 to 40% after treatment.

The objective of this pictorial essay is to depict various appearance of intrauterine adhesion which is taken by hysterosalpingography.

## Discussion

In AS, destruction of endometrium causes scar in the endometrium, followed by rapid expansion of scar tissue band or synechiae within the uterine cavity. Scarring may be minor, affecting a small area of the uterine wall, or be extensive with diffuse involvement and obliteration of the uterine cavity. Synechiae may be found anywhere in the uterine cavity. They can also involve adjacent structures, causing stenosis of tubal ostia in the corneal region or stenosis of the endocervical canal near the internal cervical canal (14).

Symptoms related to AS are as follows: infertility, recurrent pregnancy loss, menstrual irregularity specially amenorrhea, as well as cyclic pelvic pain, indicating that mensturation is occurring, but the blood cannot exit the uterus because the cervix is blocked by adhesions.

The American Fertility Society (AFS) classifies intrauterine synechia involvement by applying the combination of hysterosalpingographic, hysteroscopic and menstrual changes (10, 14) as follows: i. mild (adhesion involving one-fourth of uterine cavity), ii. moderate (adhesion involving one–half of uterine cavity), and iii. severe (adhesion involving three-fourths or more of uterine cavity). Furthermore, the stage of disease is determined by the extent of the endometrial cavity involved (adhesions throughout the uterus or just in a small area), the type of adhesions (filmy or dense) and the menstrual pattern.

AS is identified through application of the following techniques: two dimensional ultrasonography (2DUS) and 3DUS trans vaginal sonography (TVS), hystrosonography, hysterosalpingography (HSG), as well as hysteroscopy.

Hysteroscopy is the gold standard for the diagnosis of severe intrauterine adhesions (15). The result of Soares et al. study revealed that sonohysterography and HSG had a sensitivity of 75% in the detection of intrauterine adhesions and respective positive predictor values (PPVs) of 42.9 and 50% (16).

**Fig 1:**
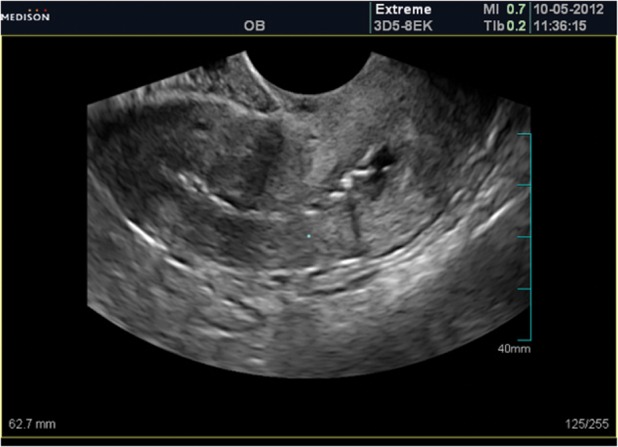
Ultrasound shows irregular endometrial contour with typical adhesion appearnace.

Figure 1 shows an ultrasound scan of a patient with AS showing a mixed picture of the endometrial line; however, the line in some parts cannot be visualized, while in other parts, the endometrium appears normal. Other appearances are adhesions, which are observed as endometrial irregularities (17). Intrauterine adhesions (IUA) appear either as eccentric echogenic or as calcificated areas in ultrasound. Endometrial thickness may be with or without focal multi cystic. If the canal is completely obliterated, there is an absent endometrial stripe in ultrasound (US) finding (4).

**Fig 2:**
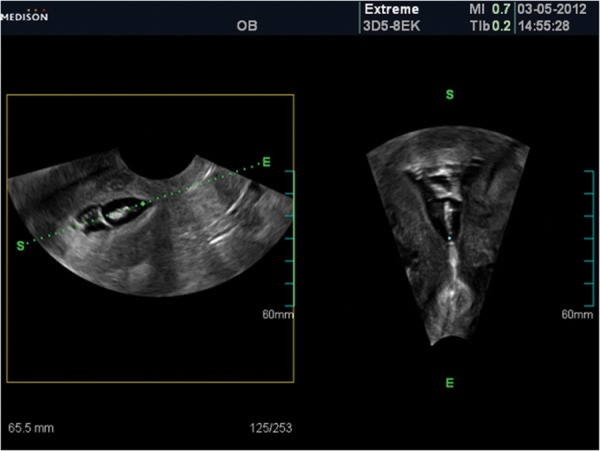
In sonohysterography there are echogenic fibrotic bands, distended endometrium side to side wall of the uterus and superior to inferior in sagital and coronal plane which is shows typic adhesion.

Three dimensional ultrasound (3DUS) is a good supplement to TVS which only obtains images in two planes. The third plane can depict the extent and location of synechiae, more thoroughly (4).

phy findings, there may be echogenic bands traversing distended endometrial canal extending side to side of uterine wall. Distention of uterine cavity with saline infusion may be done hardly (Fig 2).

The extent and location of the synechiae can be identified through HSG; however, patients may feel pain during contrast medium injection due to poor distansibility of cavity.

The radiographic appearance of intrauterine adhesions varies with the sites involved and the severity of scares (17).

**Fig 3:**
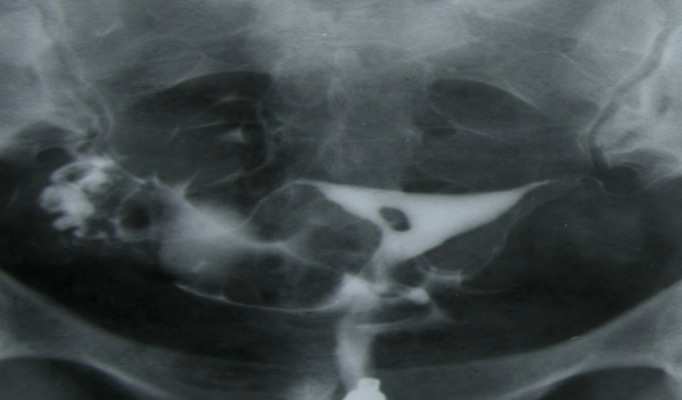
A 24 years old woman with history of three curettages. HSG detected a small filling defect with totally sharp contour and typical synechiae. The adhesion involves less than ¼ of uterine cavity.

**Fig 4:**
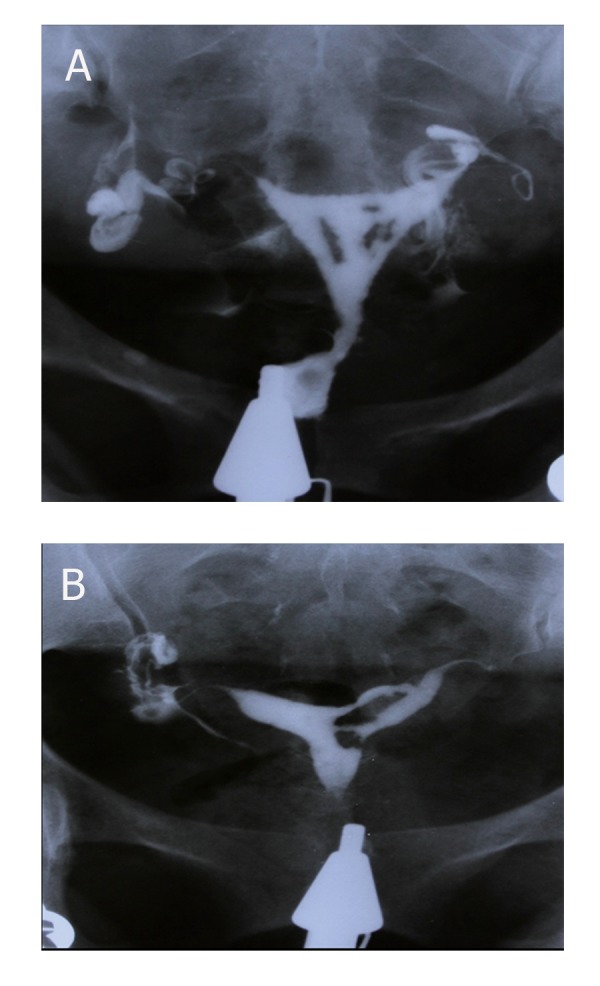
(A and B): 32 and 40 years old women with history of two curettages, each. HSG shows multiple irregular and angulated filling defects with sharp border involving ½ of uterine cavity (moderate synechiae).

**Fig 5:**
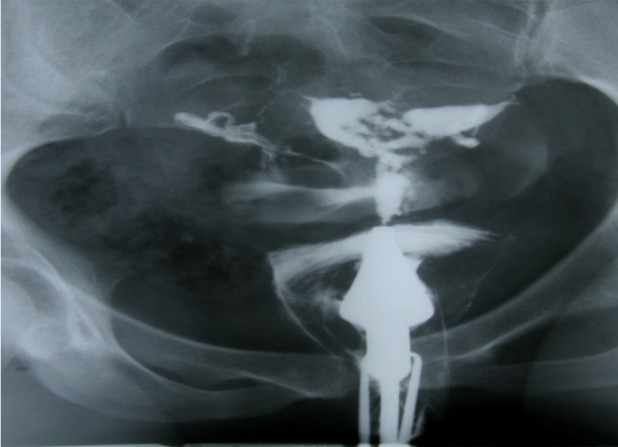
A 25-years-old woman with history of three curettages. HSG result shows that configuration of uterine cavity is totally disturbed. Multiple defects in uterine wall and cavity, considered as secondary to extensive adhesion, involve more than ¾ of the uterine cavity volume (severe synechiae).

Synechiae appears as filling defects distorting the contour of the uterine cavity; although, they typically have an irregular, multiple angulated lacunar-shaped and immobile intracavity filling defect (Figs 3-5). 

**Fig 6:**
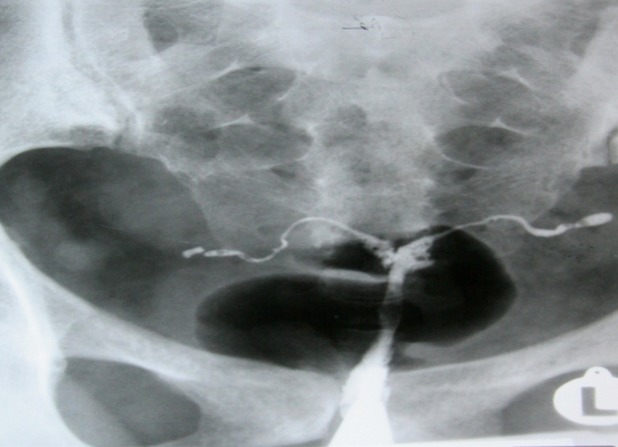
A 35 year old lady with a history of 5 year of infertility and family tuberculosis history.HSG shows Small irregular hypo plastic uterine cavity. (Dwarf uterus) Spillage of contrast media up to isthmus region of both tubes, under pressure is detected. Pipe-like appearance is detected bilaterally. All of the mentioned features could be considered in genital tuberculosis.

They are easily defined because the uterine walls are adhered, while the contrast material does not completely surround the defects (4, 17). Unlike other uterine cavity defects, increasing volumes of contrast will not obscure adhesions because there is no contrast flowing in front of or behind them (17). In cases with extensive symmetrical obliteration of the uterine cavity, sometimes, the cavity is smaller than its normal size and gives the appearance of an infantile (dwarf) uterus (Fig 6).

**Fig 7:**
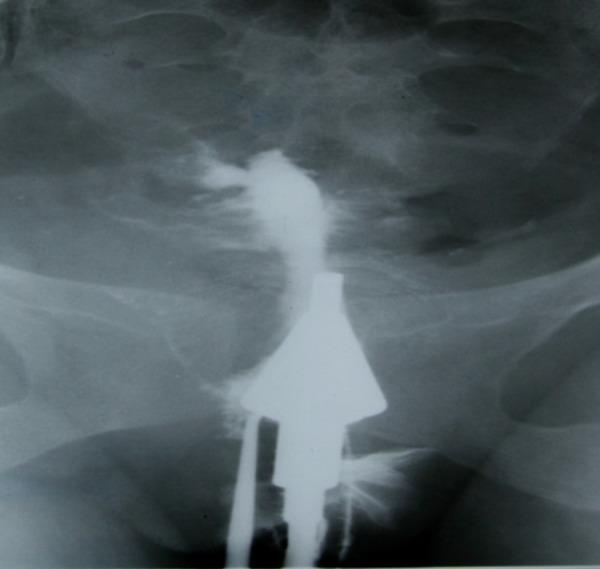
A 28-year-old woman with history of three curettages. HSG shows spindle shape cervical canal with normal appearance. There is obliteration of the isthmus and uterine cavity, considered as secondary to multiple curettages. In obtained image series, there is no filling of contrast in uterine cavity. Intravasation of contrast media into surrounded ventricular plexus is observable due to severe force of injection (Finger glove’s appearance).

In this situation, a history of previous trauma or disease, as well as clinical and sonographic signs will be helpful to diagnose adhesions (17). In 1955, Netter et al. (18) have described total obliteration of the whole uterine cavity with severe involvement. In this case, the cervical canal is observable, but there is virtually complete obliteration of the uterine cavity (Glove’s finger appearance) (Fig 7).

**Fig 8:**
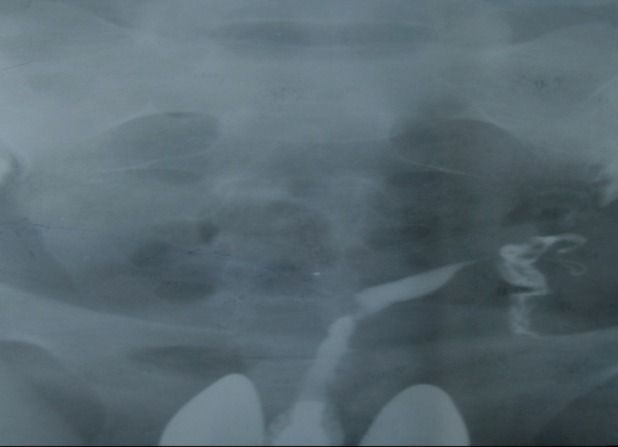
A 20 years old woman with history of one curettage. HSG shows spindle shape uterus with unicorn appearance in left pelvic cavity. Previous HSG shows normal uterus,but now, there is no filling of contrast in right corn due to adhesion. Uterus is appeared as a unicorn which is known as pseudo-unicorn uterus.

**Fig 9:**
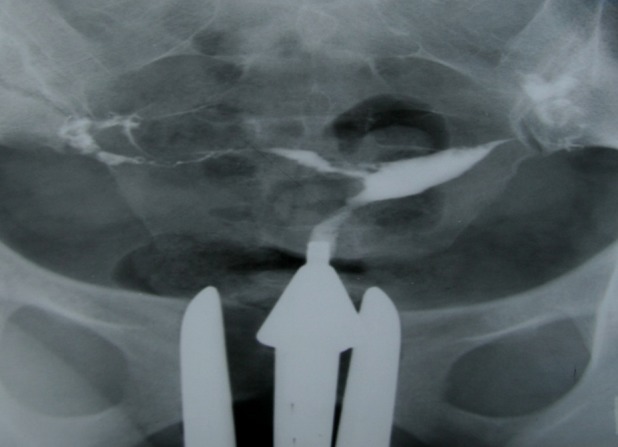
A 29-year-old woman with history of seven curettages. HSG shows irregular contour of uterine cavity and asymmetry in both corn and adhesion in right corn. Due to adhesive changes, result reveals an image mimicking bicorne or septate uterus

Asymmetrical obliteration in uterine cavity with unicorn involvement resembles unicorn (pseudo unicorn-uterus) appearances (Fig 8). Sometimes, indentation in the cavity due to synechiae resembles a septate uterus (Fig 9).

## Conclusion

**Fig 10:**
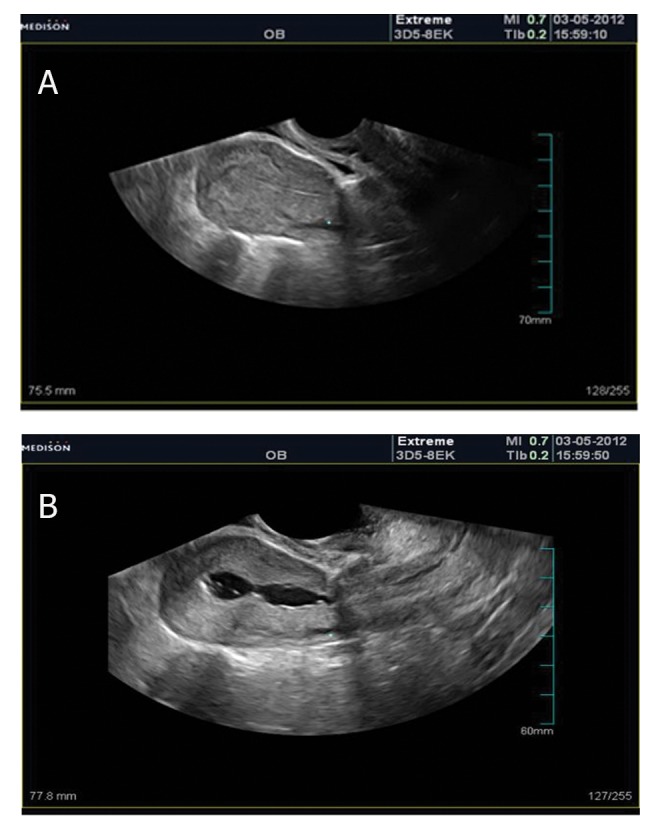
TVS shows normal appearance (A), but sonohysterography depicts IUA (B).

Ultrasound is not a reliable method for diagnosing AS compared to HSG. One study reported that transvaginal sonography showed low sensitivity and PPV for this kind of diagnosis (16) unless fluid is instilled into the uterine cavity to provide enhanced endometrial visualization during transvaginal ultrasound examination (Fig 10), which it clarifies the reason behind the falsenegative of sonography findings in depicting IUA.

The gold standard is to look directly at the uterine cavity and scar tissue using hysteroscopy (12). However, HSG reveals the extent of the scar formation, while suggesting the presence of intrauterine adhesions (19). Furthermore, as there is a high correlation between the diagnosis by hysteroscopy and HSG, hysterosalpingography is known as one of the appropriate imaging technique (3). HSG is commonly used as a first-line tool in the diagnosis of IUA because it is simple, safe, cost effective, sensitive, and minimally invasive procedure, allowing the visualization of the uterine cavity and tubal patency (14).
